# International Circumpolar Surveillance System for Invasive Pneumococcal Disease, 1999–2005

**DOI:** 10.3201/eid1401.071315

**Published:** 2008-01

**Authors:** Michael G. Bruce, Shelley L. Deeks, Tammy Zulz, Dana Bruden, Christine Navarro, Marguerite Lovgren, Louise Jette, Karl Kristinsson, Gudrun Sigmundsdottir, Knud Brinkløv Jensen, Oistein Lovoll, J. Pekka Nuorti, Elja Herva, Anders Nystedt, Anders Sjostedt, Anders Koch, Thomas W. Hennessy, Alan J. Parkinson, the International Circumpolar Surveillance System for Invasive Pneumococcal Disease Working Group

**Affiliations:** * Centers for Disease Control and Prevention, Anchorage, Alaska, USA; † Public Health Agency of Canada, Ottawa, Ontario, Canada; ‡ National Centre for Streptococcus, Edmonton, Alberta, Canada; § Quebec Public Health Laboratory, Ste. Anne-de-Bellevue, Quebec, Canada; ¶ Landspitali University Hospital, Reykjavik, Iceland; # Institution of the Chief Medical Officer, Nuuk, Greenland; ** Norwegian Institute of Public Health, Oslo, Norway; †† National Public Health Institute, Helsinki, Finland; ‡‡ National Public Health Institute, Oulu, Finland; §§ Sunderby Hospital, Lulea, Sweden; ¶¶ Umea University, Umea, Sweden; ## Statens Serum Institut, Copenhagen, Denmark; 1 Current affiliation: National Centre for Immunisation, Research and Surveillance, Westmead, New South Wales, Australia

**Keywords:** Streptococcus pneumoniae, invasive pneumococcal disease, IPD, surveillance, PCV7, serotype replacement, indigenous, Alaska Native, circumpolar, arctic, research

## Abstract

The International Circumpolar Surveillance System is a population-based surveillance network for invasive bacterial disease in the Arctic. The 7-valent pneumococcal conjugate vaccine (PCV7) was introduced for routine infant vaccination in Alaska (2001), northern Canada (2002–2006), and Norway (2006). Data for invasive pneumococcal disease (IPD) were analyzed to identify clinical findings, disease rates, serotype distribution, and antimicrobial drug susceptibility; 11,244 IPD cases were reported. Pneumonia and bacteremia were common clinical findings. Rates of IPD among indigenous persons in Alaska and northern Canada were 43 and 38 cases per 100,000 population, respectively. Rates in children <2 years of age ranged from 21 to 153 cases per 100,000 population. In Alaska and northern Canada, IPD rates in children <2 years of age caused by PCV7 serotypes decreased by >80% after routine vaccination. IPD rates are high among indigenous persons and children in Arctic countries. After vaccine introduction, IPD caused by non-PCV7 serotypes increased in Alaska.

## Abstract 2

Disease rates are high among indigenous persons in Arctic countries, and PCV7 has resulted in decreased rates in North American children.

The International Circumpolar Surveillance (ICS) project was established in 1999 to create an infectious disease surveillance network throughout Arctic countries and territories. The project initially focused on invasive bacterial diseases caused by *Streptococcus pneumoniae*, *Haemophilus influenzae*, *Neisseria meningitidis*, and groups A and B streptococci. In 1999, the project integrated prospective population-based surveillance data for invasive pneumococcal disease (IPD) from the US Arctic (Alaska) and northern Canada (*1*). Subsequently, the ICS network expanded to include Greenland in 2000, Iceland, Norway, and Finland in 2001, and northern Sweden in 2003. All northern circumpolar countries (north of latitude 60°N), with the exception of Russia, participate in ICS.

IPD was of interest to the ICS in part because it is one of the leading causes of pneumonia and meningitis among indigenous persons of the circumpolar north. Incidence rates of IPD are higher among indigenous persons than among nonindigenous persons (*2*–*6*). The proportion of indigenous persons in Alaska and northern Canada <5 years of age is 11% and 12%, respectively. In Alaska, the 7-valent pneumococcal conjugate vaccine (PCV7) was made available to all children through a statewide immunization program. Data indicate that 92.6% of indigenous children and 64.6% of non-Hispanic white children in Alaska received >3 doses of PCV7 from July 2003 through June 2004, respectively. Routine use of PCV7 began in some areas of northern Canada in 2002. Other areas of northern Canada implemented PCV7 programs during 2003–2006. Scant data exist on vaccine coverage in northern Canada. However, neighboring Canadian provinces outside the ICS network, such as Alberta, have vaccine coverage rates >90% (www.phac-aspc.gc.ca/publicat/ccdr-rmtc/05pdf/cdr3106.pdf). Routine use of PCV7 began in Norway in 2006. PCV7 is not currently included in the routine infant immunization schedule in Greenland, Iceland, northern Sweden, or Finland.

We analyzed IPD data collected from January 1999 through December 2005. The purpose of the study was to determine rates of disease by country, common clinical findings, risk factors, serotype distribution, antimicrobial drug susceptibility patterns, and changes in disease rates with vaccine use.

## Methods

In the participating regions, clinical laboratories send *S*. *pneumoniae* isolated from a normally sterile site to regional reference laboratories (Table 1). Reference laboratories confirm the identity, determine the serotype, and test for antimicrobial drug susceptibility of each isolate. Laboratory, demographic, and clinical data are collected for each invasive case of *S*. *pneumoniae*, and these data are forwarded to the Arctic Investigations Program (AIP) of the Centers for Disease Control and Prevention (CDC) in Anchorage, Alaska, the coordinating center for ICS.

**Table 1:** Demographics of countries participating in the study

Characteristic	Alaska	Northern Canada	Greenland	Iceland	Norway	Northern Sweden	Finland
Mean population	641,720	132,956	56,617	288,035	4,565,943	252,729	5,215,791
% Indigenous	19	59	Unknown	Unknown	<1	<5	<1
Region size, km^2^	1,518,807	4,506,600	2,131,863	102,968	323,760	160,580	339,290
No. participating laboratories	23	14	15	10	33	1	23
Location of reference laboratories	Anchorage	Edmonton, Montreal, Winnipeg	Nuuk, Copenhagen	Reykjavik	Oslo, Tromso	Stockholm	Oulu

Population distribution and land area vary widely among participating regions. Populations range from ≈57,000 persons in Greenland to >5 million persons in Finland. Land areas range from 102,968 km^2^ for Iceland to 4,506,600 km^2^ for northern Canada (Table 1). Regions with the largest land areas (Alaska, Greenland, and northern Canada) have populations scattered in remote, small communities often not connected by road to urban centers. Remote populations in Scandinavia and Iceland are better connected to larger urban communities.

A case-patient with IPD was defined as a resident of the surveillance area from whom *S*. *pneumoniae* was isolated from a normally sterile site, including blood, cerebrospinal fluid, pleural fluid, peritoneal fluid, or joint fluid. Population denominator data for the regions were obtained from the Statistics Canada Website (www.statcan.ca), the Alaska Department of Labor and Workforce Development Website (www.labor.state.ak.us), the Statistics Greenland Website (www.statgreen.gl), the Statistics Iceland Website (www.hagstofa.is), the Statistics Norway Website (www.ssb.no), the Statistics Sweden Website (www.scb.se), and the Statistics Finland Website (www.stat.fi). This study covers a 7-year surveillance period from January 1999 through December 2005.

### Epidemiologic Data

#### Alaska and Northern Canada

Initial identification of a case of IPD results in a report to local public health personnel who complete standardized data collection forms (bacterial disease surveillance form [BDSF]) that include demographic, clinical, and risk factor information, and pertinent immunization history (www.cdc.gov/ncidod/aip/research/ics/forms.html). All laboratory and case-related data are forwarded without identifiers by fax and mail to the ICS coordinator at AIP in Anchorage, Alaska, where they are entered into a database and analyzed.

#### Greenland, Iceland, Norway, Northern Sweden, and Finland

End of year summary data are submitted electronically to the ICS coordinator at AIP in Anchorage, where they are entered into a database. Greenland and Iceland use the BDSF. Norway, northern Sweden, and Finland use other instruments.

### Laboratory Data

Isolates were serotyped by the Quellung reaction (Alaska, northern Canada, Greenland, Norway, and northern Sweden), counter-immunoelectrophoresis (Finland), or coagglutination (Iceland). Antimicrobial drug susceptibility testing was performed by microbroth dilution (Alaska and northern Canada), agar dilution (Greenland and Finland), or disk diffusion (Iceland, Norway, and northern Sweden). A laboratory quality control program has been in place since the program’s inception (*7*).

### Statistical Analysis

Data were double-entered into Paradox version 10.0 (Corel, Ottawa, Ontario, Canada), and analyzed by using EpiInfo version 6.04b (CDC, Atlanta, GA, USA), SAS version 8.0 (SAS Institute, Cary, NC, USA), and StatXact version 6.0 (Cytel Corporation, Cambridge, MA, USA). For Alaska, we compared disease rates by using 1999–2000 as the baseline period and 2001–2005 as the postvaccine period. For northern Canada, we used 1999–2002 as the baseline period and 2003–2005 as the vaccine implementation period. Standardized incidence rates were reported by using World Health Organization 2000 population standard and the age groups of <1, 2–19, 20–64, and >65 years. Statistical differences in rates between periods and between countries were assessed by using a 2-sample Poisson test. Trends in IPD incidence rates among children <2 years of age were assessed by using Poisson regression; p values are exact when appropriate.

## Results

### Descriptive Epidemiology

Over the 7-year surveillance period, 11,244 cases of IPD were detected among the 7 participating countries; 4,921 (53%) were in male patients. Of the 5,896 case-patients for whom outcome was reported, 569 died (case-fatality rate 10%). Median age of case-patients was 57 years and varied by country (Table 2). Among countries that did not use PCV7 during the study period, overall crude IPD incidence rates ranged from 11.6 in northern Sweden (age standardized rate 9.1) to 21.0 in Norway (age standardized rate 16.2) (Table 3).

**Table 2:** Characteristics of persons with invasive pneumococcal disease, by country*

Characteristic	Alaska, 1999–2005, n = 769	Northern Canada, 1999–2005, n = 251	Greenland, 2000–2005, n = 69	Iceland, 2000–2005, n = 274	Norway, 2000–2005, n = 5,744	Northern Sweden, 2003–2005, n = 88	Finland, 2000–2005, n = 4,049
Median age (range)	41.6 (1 mo–100 y)	30.2 (0 mo– 83 y)	44.7 (0 mo–91 y)	53.2 (1 mo–98 y)	63.2 (0 mo–99 y)	65.8 (9 mo–98 y)	54.2 (0 mo–100 y)
No. males (%)	423 (55)	149 (60)	37 (54)	145 (53)	2,856 (50)	40 (45)	2,271 (56)
No. indigenous (%)	372 (48)	191 (84)†	NA	NA	NA	NA	NA
No. hospitalized (%)	585 (77)‡	201 (87)‡	62 (100)‡	NA	5,567 (99)‡	NA	NA
Duration of hospitalization, d, median (minimum–maximum)	4 (0–188)	5 (0–77)	9 (0–131)	NA	NA	NA	NA
No. deaths (%)	96 (13)§	11 (5)§	13 (20)§	30 (27)§	419 (9)§	NA	NA

*NA, not available. †Ethnicity data missing from 24 (northern Canada) cases. Denominator = 227. ‡Hospitalization data missing for 9 (Alaska), 21 (northern Canada), 7 (Greenland), and 127 (Norway) cases. Denominators are 760, 230, 62, and 5,617, respectively. §Death information missing for 10 (Alaska), 21 (northern Canada), 3 (Greenland), 161 (Iceland), and 1,1016 (Norway) cases. Denominators are 759, 230, 66, 113, and 4,728, respectively.

**Table 3:** Annualized crude and standardized incidence rates (per 100,000 persons) of IPD by countries not using 7-valent pneumococcal conjugate vaccine*

Statistic or age group	Greenland (2000–2005)	Iceland (2000–2005)	Norway (2000–2005)	Northern Sweden (2003–2005)	Finland (2000–2005)
Total no. cases	69	274	5,744	88	4,049
Age-specific annualized incidence rates (no. cases)					
<2 y	77.4 (8)	89.8 (45)	50.0 (355)	21.1 (3)	52.3 (367)
2–19 y	4.8 (5)	6.8 (32)	4.9 (312)	0.0 (0)	5.0 (346)
20–64 y	25.5 (53)	8.9 (90)	14.5 (2,352)	9.3 (41)	10.9 (2,057)
>65 y	16.6 (3)	53.1 (107)	66.7 (2,725)	30.9 (44)	26.6 (1,279)
Crude annualized incidence (all ages)	20.3	15.9	21.0	11.6	12.9
Annualized age standardized incidence†	19.8	14.6	16.2	9.1	11.6

*IPD, invasive pneumococcal disease, †Rates adjusted to 2000 World Health Organization world standard population estimates.

Annualized age-specific incidence rates over the entire surveillance period were highest in children <2 years of age and the elderly (Tables 3, 4). Within each country, rates were highest in children <2 years of age except for Norway and northern Sweden, where rates were slightly higher in the elderly.

**Table 4:** Rates/100,000 cases of IPD in Alaska and Northern Canada before and after introduction of PCV7*

Group	Alaska				Northern Canada		
	Prevaccine (1999–2000)	Postvaccine (2001–2005)	p value		Prevaccine (1999–2002)	Vaccine implementation (2003–2005)	p value
Total no. cases	257	512	NA		165	86	NA
All ages, y	20.6 (257)	15.8 (512)	0.0004		31.0 (165)	21.6 (86)	0.007
<2	173.5 (69)	79.2 (82)	<0.0001		185.6 (36)	110.0 (16)	0.10
2–19	10.7 (40)	6.6 (64)	0.02		22.9 (41)	9.7 (13)	0.009
20–64	13.7 (104)	14.1 (278)	0.82		23.8 (74)	18.8 (44)	0.24
>65	57.9 (44)	44.5 (88)	0.17		64.4 (14)	73.5 (12)	0.84
Indigenous, all ages	56.0 (133)	38.1 (239)	0.0003		44.2 (134)	25.0 (57)	0.0005
<2 y	440.6 (47)	177.5 (50)	<0.0001		229.3 (33)	92.6 (10)	0.01
Nonindigenous, all ages	12.3 (124)	10.4 (273)	0.13		9.6 (20)	10.2 (16)	0.86
<2 y	75.7 (22)	42.5 (32)	0.05		65.4 (3)	87.2 (3)	1.00
PCV 7 serotypes (4, 6B, 9V, 14, 18C, 19F, 23F)†							
All ages, y	9.6 (120)	3.4 (110)	<0.0001		12.6 (67)	3.8 (15)	<0.0001
<2	128.3 (51)	15.5 (16)	<0.0001		128.9 (25)	20.6 (3)	0.0008
2–19	5.6 (21)	1.6 (16)	0.0003		8.4 (15)	1.5 (2)	0.01
20–64	4.1 (31)	2.8 (55)	0.09		7.1 (22)	1.7 (4)	0.005
≥65	22.4 (17)	11.6 (23)	0.05		23.0 (5)	36.8 (6)	0.55
Indigenous, all ages	24.9 (59)	4.9 (31)	<0.0001		17.1 (52)	3.5 (8)	<0.0001
<2 y	318.7 (34)	21.3 (6)	<0.0001		159.8 (23)	9.3 (1)	0.0001
Nonindigenous, all ages	6.0 (61)	3.0 (79)	<0.0001		6.2 (13)	3.2 (5)	0.24
<2 y	58.5 (17)	13.3 (10)	<0.0001		43.6 (2)	29.1 (1)	1.00
Non-PCV7 serotypes†							
All ages, y	8.3 (104)	10.5 (341)	0.04		17.1 (91)	16.8 (67)	0.94
<2	27.7 (11)	59.0 (61)	0.03		41.2 (8)	75.6 (11)	0.25
2–19	3.5 (13)	4.1 (40)	0.65		14.5 (26)	7.4 (10)	0.09
20–64	7.3 (55)	9.6 (189)	0.07		15.4 (48)	16.7 (39)	0.75
>65	32.9 (25)	25.8 (51)	0.31		41.4 (9)	36.8 (6)	1.00
Indigenous, all ages	20.2 (48)	29.8 (187)	0.01		25.4 (77)	20.2 (46)	0.24
<2 y	65.6 (7)	145.6 (41)	0.05		48.6 (7)	74.1 (8)	0.44
Nonindigenous, all ages	5.5 (56)	5.9 (154)	0.71		2.9 (6)	7.0 (11)	0.09
<2 y	13.8 (4)	26.6 (20)	0.26		21.8 (1)	58.1 (2)	0.58
Penicillin nonsusceptible IPD, all serotypes‡							
All ages	4.0 (50)	2.1 (68)	0.0004		1.5 (8)	0.8 (3)	0.37
<2 y	62.9 (25)	21.3 (22)	<0.0001		10.3 (2)	0.0 (0)	0.51
Cotrimoxazole nonsusceptible IPD, all serotypes‡							
All ages	5.6 (70)	3.0 (96)	0.0003		2.6 (14)	1.8 (7)	0.50
<2 y	90.5 (36)	25.1 (26)	<0.0001		15.5 (3)	13.7 (2)	1.00

*IPD, invasive pneumococcal disease, PCV7, 7-valent pneumococcal conjugate vaccine; NA, not available. Values in parentheses are no. cases. †Serotype available for 675 (88%) of 769 Alaska isolates and 240 (96%) of 251 Northern Canada isolates. ‡Antimicrobial drug susceptibility available for 677 (88%) of 769 Alaska isolates and 236 (94%) of 251 northern Canada isolates.

Race and ethnicity data were only available from Alaska and northern Canada. Over the entire surveillance period, annualized rates of disease were higher in Alaska Native (indigenous) persons (43.1 cases/100,000/year) than in nonindigenous persons (9.8 cases) (relative risk [RR] 4.4, 95% confidence interval [CI] 3.8–5.1). Increased risk for IPD was similar in northern Canada (RR 3.6, 95% CI 2.6, 5.2) with an annualized overall rate of 36.0 cases/100,000 among Canadian indigenous persons and 9.9 cases/100,000 among nonindigenous persons. Among children <2 years of age, increased RR for indigenous versus nonindigenous children was 6.5 in Alaska (95% CI 4.5, 9.4; indigenous 249.9 cases/100,000/year, nonindigenous 38.3 cases/100,000/year) and 2.3 in Canada (95% CI 1.0, 5.2; indigenous 170.7 cases/100,000/year, nonindigenous 74.7 cases/100,000/year).

### Incidence Rates (All Serotypes) in Countries Using PCV7

In Alaska, incidence of IPD among all age groups decreased from 20.6 cases in the prevaccine period (1999–2000) to 15.8 cases/100,000 persons in the postvaccine period (2001–2005; p = 0.0004). Among Alaskan children <2 years of age, incidence of IPD decreased from 173.5 cases in the pre–conjugate vaccine period to 79.2 cases/100,000 children in the postvaccine period (p<0.0001). Similarly, in northern Canada, incidence of IPD among all age groups decreased from 31.0 cases/100,000 persons in the prevaccine period (1999–2002) to 21.6 cases/100,000 persons in the vaccine implementation period (2003–2005; p = 0.007) (Table 4). Among northern Canadian children <2 years of age, incidence of IPD had been decreasing in the 2 years before PCV7 use (Figure). The incidence of IPD decreased from 185.6 cases/100,000 children in the prevaccine period (1999–2002) to 110.0 cases/100,000 children in the vaccine implementation period (2003–2005), However, this decrease was not statistically significant (p = 0.10) (Table 4).

**Figure:**
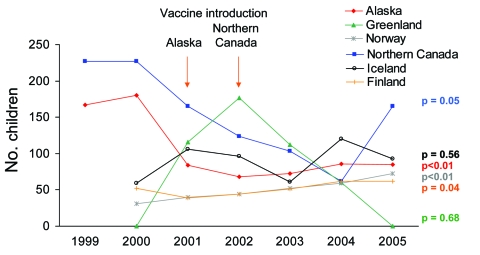
Annual invasive pneumococcal disease rates among children <2 years of age by International Circumpolar Surveillance System member country, 1999–2005. The p values are for trend.

In Alaska, incidence of IPD among all indigenous persons decreased from 56.0 cases/100,000 persons in the pre–conjugate vaccine period (1999–2000) to 38.1 cases/100,000 persons in the postvaccine period (2001–2005; p = 0.0003). Among Alaskan indigenous children <2 years of age, incidence decreased from 440.6 in the pre–conjugate vaccine period to 177.5 in the postvaccine period (p<0.0001). Similarly, in northern Canada, incidence of IPD among indigenous persons decreased from 44.2 cases/100,000 persons in the prevaccine period (1999–2002) to 25.0 cases/100,000 persons in the vaccine implementation period (2003–2005; p = 0.0005). Among Canadian indigenous children <2 years of age, incidence decreased from 229.3/100,000 persons in the prevaccine period to 92.6/100,000 persons in the vaccine implementation period (2003–2005; p = 0.01) (Table 4).

### PCV7 Serotype–specific Incidence Rates in Countries Using PCV7

In Alaska, the incidence of serotypes contained in PCV7 in all age groups decreased from 9.6 cases/100,000 persons in the pre–conjugate vaccine period (1999–2000) to 3.4 cases/100,000 persons in the postvaccine period (2001–2005; p<0.0001). PCV7 serotype–specific incidence among Alaska children <2 years decreased from 128.3 cases/100,000 persons in the pre–conjugate vaccine period to 15.5 cases/100,000 persons in the postvaccine period (p<0.0001). Among older age groups in Alaska (2–19, 20–64, and >65 years), decreases in PCV7 serotype–specific incidence also occurred. However, this decrease was not statistically significant in persons 20–64 years of age (Table 4). In northern Canada, incidence of serotypes in PCV7 in all age groups decreased from 12.6 cases/100,000 persons in the pre–conjugate vaccine period (1999–2002) to 3.8 cases/100,000 persons in the vaccine implementation period (2003–2005; p<0.0001) (Table 4). PCV7 serotype–specific incidence among northern Canadian children <2 years of age decreased from 128.9 cases/100,000 children in the prevaccine period to 20.6 cases/100,000 children in the vaccine implementation period (p = 0.0008; Table 4). Decreases in age-specific incidence of PCV7 disease also occurred among older age groups in northern Canada except among persons >65 years of age (Table 4). Among indigenous persons in Alaska and northern Canada, PCV7 serotype–specific incidence showed a statistically significant decrease from the prevaccine period to the postvaccine implementation period (Table 4).

In Alaska, non-PCV7 serotype–specific incidence among all age groups increased from 8.3 cases/100,000 persons in the pre–conjugate vaccine period (1999–2000) to 10.5 cases/100,000 persons in the postvaccine period (2001–2005; p = 0.04). Non-PCV7 serotype–specific incidence among Alaskan children <2 years of age increased from 27.7 cases/100,000 children in the pre–conjugate vaccine period to 59.0 cases/100,000 children in the postvaccine period (p = 0.03). In northern Canada, non-PCV7 serotype–specific incidence among all age groups remained relatively stable at 17.1 cases/100,000 persons in the prevaccine period (1999–2002) and 16.8 cases/100,000 persons in the vaccine implementation period (2003–2005; p = 0.94) (Table 4). Non-PCV7 serotype–specific incidence among northern Canadian children <2 years of age increased from 41.2 cases/100,000 children in the pre–conjugate vaccine period (8 cases) to 75.6 cases/100,000 children in the vaccine implementation period (11 cases). However, this increase was not statistically significant (p = 0.25; Table 4). Among indigenous persons in Alaska, non-PCV7 serotype–specific incidence demonstrated a statistically significant increase from the prevaccine to the postvaccine period. However, this trend was not seen among Canadian indigenous persons (Table 4).

### Incidence Rates (All Serotypes) in Countries Not Using PCV7

IPD rates among children <2 years of age increased in Norway and Finland (p<0.01 and p = 0.04, respectively). A slight increase was also seen in Iceland, but this increase was not statistically significant (p = 0.56). Because of low numbers of cases, rates in Greenland were unstable but showed no statistically significant change (Figure).

### Serotype Distribution

Several serotypes were common among Arctic countries participating in ICS, including 14, 4, 7F, and 6B. Among the 4 most prevalent serotypes in the Arctic, 3 are found in PCV7.

In Alaska, before vaccine use, the 5 most common serotypes were 14 (17%), 4 and 7F (9%), 9V (8%), 19F (6%), and 6B (6%). In the prevaccine period among children <2 years of age, 82% of serotypes in Alaska were in PCV7. After introduction of PCV7 (2001–2005), 21% of serotypes were in PCV7 (Table 5), and among all ages 19A (11%) is now the most prevalent serotype, followed by 4 (8%), 12F (8%), 3/7F/8 (7%), and 14 (6%).

**Table 5:** Most prevalent serotypes in 6 countries reporting *Streptococcus pneumoniae* type to ICS and proportion of isolates covered by PCV7 and PCV13 vaccines*

Rank	Alaska			Canada		Greenland, n = 60	Iceland, n = 269	Norway, n = 291	Finland, n = 3,947
	Pre-PCV7, 1999–2000, n = 224	Post-PCV7, 2001–2005, n = 453		Pre-PCV7, 1999–2002, n = 158	Post-PCV7, 2003–2005, n = 82				
1	14 (17%)	19A (11%)		1 (34%)	1 (24%)	1 (22%)	7 (20%)	4, 14 (18%)	14, 4 (12%)
2	4, 7F (9%)	4 (8%)		14 (11%)	8 (11%)	12F (15%)	14 (12%)	9 (11%)	9V (8%)
3	9V (8%)	12F (8%)		4 (9%)	3 (7%)	4 (12%)	23 (12%)	6 (9%)	3, 23F, 7F (7%)
4	19F (6%)	3, 7F, 8 (7%)		8 (8%)	10A, 18C, 22F (6%)	22F (8%)	19 (10%)	23 (8%)	6B (6%)
5	6B (6%)	14 (6%)		6B, 9V (6%)	6B (5%)	3 (7%)	9 (10%)	7 (7%)	19A, 19F (4%)
Proportion of serotyped isolates covered by PCV7 and PCV13 vaccines (<2 y of age)									
PCV7	82% (51/62)	21% (16/77)		76% (25/33)	21% (3/14)	50% (3/6)	51% (23/45)	37% (10/27)	NA
PCV13	92% (57/62)	57% (44/77)		94% (31/33)	43% (6/14)	83% (5/6)	60% (27/45)	56% (15/27)	NA

*ICS, International Circumpolar Surveillance; PCV7, 7-valent pneumococcal conjugate vaccine (serotypes 4, 6B, 9V, 14, 18C, 19F, and 23F); PCV13, 13-valent pneumococcal conjugate vaccine (7 PCV7sero types plus 1, 3, 5, 6A, 7F, and 19A); NA, not available.

In northern Canada, the 5 most prevalent serotypes before PCV7 use were 1 (34%), 14 (11%), 4 (9%), 8 (8%), and 6B/9V (6% each). In the prevaccine period, among children <2 years of age, 76% of serotypes in northern Canada were in PCV7. During the vaccine implementation period (2003–2005), 21% of serotypes in northern Canada were in PCV7 (Table 5). Serotype distribution (all age groups combined) has changed; however, serotype 1 (24%) continues to be the most prevalent serotype, followed by 8 (11%), 3 (7%), 10A/18C/22F (6%), and 6B (5%). Two regions in northern Canada had outbreaks of serotype 1 during the surveillance period, which may have affected relative frequencies of serotypes (*8*–*10*).

### Antimicrobial Drug Resistance

Alaska had the highest proportion of isolates nonsusceptible to antimicrobial drugs among ICS regions reporting these data. With use of PCV7, the proportion of isolates nonsusceptible to penicillin among children <2 years of age decreased from 40% to 29% and from 6% to 0% (prevaccine to postvaccine period) in Alaska and northern Canada, respectively (Table 6). Rates of IPD with penicillin-nonsusceptible isolates decreased from 62.9 cases/100,000 children to 21.3 cases/100,000 children (p<0.0001) in Alaska, and rates decreased from 10.3 cases/100,000 children to 0 cases/100,000 children (p = 0.51) in northern Canada (Table 4). However, in 2 countries currently not using PCV7, comparison of 2 periods (2000–2002 and 2003–2005) showed that rates of IPD with penicillin-nonsusceptible isolates increased from 0.6 cases to 1.7 cases/100,000 persons (p = 0.04 for all ages, Iceland), and from 0.5 to 1.0 (p<0.0001, all ages. Finland). In both of these countries, the proportion of isolates that were nonsusceptible to penicillin also increased (Iceland, 5% [5/106] to 12% [15/130], p = 0.05; Finland, 4% [82/1,850] to 7% [154/2,199]; p<0.01).

**Table 6:** Proportion of isolates with nonsusceptibility to antimicrobial drugs in countries in the ICS reporting an antibiogram*

Antimicrobial drug	Age group, y	Alaska			Northern Canada		Iceland, % (n/N)	Northern Sweden, % (n/N)
		Pre-PCV7, % (n/N)	Post-PCV7, % (n/N)		Pre-PCV7, % (n/N)	Post-PCV7, % (n/N)		
Cotrimoxazole	<2	58 (36/62)	34 (26/77)		9 (3/33)	17 (2/12)	35 (13/37)	100 (1/1)
	All ages	31 (70/224)	21 (96/453)		9 (14/158)	9 (7/78)	18 (43/234)	12 (3/25)
Erythromycin	<2	42 (26/62)	13 (10/77)		0 (0/33)	8 (1/13)	26 (10/38)	0 (0/2)
	All ages	21 (47/223)	8 (37/452)		1 (1/157)	5 (4/79)	9 (21/235)	6 (3/53)
Ceftriaxone†	<2	23 (14/62)	5 (4/77)		6 (2/33)	0 (0/12)	0 (0/10)	NA
	All ages	11 (25/224)	1 (6/453)		4 (7/159)	0 (0/80)	0 (0/39)	NA
Penicillin‡	<2	40 (25/62)	29 (22/77)		6 (2/33)	0 (0/13)	13 (5/38)	0 (0/1)
	All ages	22 (50/224)	15 (68/453)		5 (8/159)	4 (3/81)	8 (20/236)	2 (1/52)

*ICS, International Circumpolar Surveillance; PCV7, 7-valent pneumococcal conjugate vaccine; NA, not available. †Greenland reported nonsusceptibility of 0% (0/38) to ceftriaxone among all ages. ‡Greenland reported nonsusceptibility of 0% (0/41) and Finland reported nonsusceptibility of 6% (236/4,049) to penicillin among all ages. Finland reported nonsusceptibility of 9% (33/367) to penicillin among cases <2 y of age.

Since routine use of PCV7, the proportion of isolates nonsusceptible to cotrimoxazole, erythromycin, ceftriaxone, and penicillin decreased in Alaska. The same trend was not observed in northern Canada, where rates of antimicrobial drug resistance were much lower than in Alaska (Table 6).

### Clinical Findings

Data on clinical findings were available for Alaska, northern Canada, Greenland, and Norway. Bacteremic pneumonia was the most common clinical finding (range 45%–65%), followed by bacteremia alone (range 16%–24%) and bacteremic meningitis (Table 7). Limited clinical data were available for Iceland and northern Sweden but were not included. Clinical data were not available for Finland.

**Table 7:** Clinical findings for invasive pneumococcal disease

Findings	Alaska, 1999–2005, no. (%)	Northern Canada, 1999–2005, no. (%)	Greenland, 2000–2005, no. (%)	Norway, 2000–2005, no. (%)
Pneumonia with bacteremia	466 (61)	162 (65)	36 (52)	2,598 (45)
Sepsis	154 (20)	41 (16)	14 (20)	1,404 (25)
Bacteremia	20 (3)	13 (5)	0	864 (15)
Meningitis with bacteremia	53 (7)	16 (6)	14 (20)	454 (8)
Other*	76 (10)	19 (8)	5 (7)	405 (7)
Total	769 (100)	251 (100)	69 (100)	5,725 (100)

*Empyema, cellulitis, necrotizing fasciitis, septic arthritis.

### Risk Factors and Medical Conditions in Persons ≥18 Years of Age

Data on risk factors and medical conditions were available for Alaska and northern Canada. Among adults with a diagnosis of IPD in the North American Arctic, 40%–44% had a history of smoking, 37%–39% had a history of alcohol abuse, and 19%–27% had a history of chronic lung disease (Table 8).

**Table 8:** Risk factors and medical conditions in persons >18 years of age with invasive pneumococcal disease*

Factor or condition	Alaska, 1999–2005, no. (%)	Northern Canada, 1999–2005, no. (%)
Cigarette smoking	223 (44)	54 (40)
Alcohol abuse†	201 (39)	50 (37)
Chronic lung disease/asthma	139 (27)	26 (19)
Diabetes mellitus	71 (14)	22 (16)
Immunosuppressive therapy	35 (7)	5 (4)
Injection drug use	11 (2)	3 (2)
Asplenia	9 (2)	4 (3)
Total	509 (100)	135 (100)

*Risk factors and medical conditions are not mutually exclusive. Each case may have >1 condition reported. Data were not available for Greenland, Iceland, Norway, northern Sweden, or Finland. †Alcohol abuse was noted in the chart.

## Discussion

Our data show that cases of IPD continue to occur throughout Arctic countries with highest rates among children <2 years of age, adults >65 years of age, and indigenous persons of the North American Arctic. The rate of IPD with PCV7 serotypes in children <2 years of age decreased dramatically after routine vaccination in Alaska (128.3 to 15.5/100,000) and northern Canada (128.9 to 20.6/100,000). However, in Alaska, rates of non-PCV7 serotypes among children <2 years of age increased (27.7 to 59.0/100,000) during the same period. Although PCV7 serotype–specific IPD rates decreased among children <2 of age in countries that have implemented use of PCV7, overall rates of IPD among indigenous North American children remain high at 177.5 cases/100,000 and 92.6 cases/100,000 in Alaska and northern Canada, respectively, showing persistent health disparities.

Consistent with earlier studies, IPD rates were highest in the prevaccine period among indigenous persons in Alaska and northern Canada and high among children <2 years of age and the elderly (*1*,*5*,*11*–*18*). The relatively higher rates among children <2 years of age in Alaska and northern Canada (compared with other circumpolar countries) are primarily caused by increased rates in large indigenous populations (19% and 59% of the total population in Alaska and northern Canada, respectively), which tend to live under conditions of crowding, increasing environmental stress, and lower socioeconomic status.

IPD rates in the prevaccine period among indigenous children ranged from 229/100,000 (northern Canada) to 441/100,000 (in Alaska). These rates that are 4–6 times higher than those found among nonindigenous children in those regions. Indigenous persons in the circumpolar north have been shown to have high rates of IPD (*1*,*2*,*5*,*15*,*19*–*21*), as have other indigenous groups such as Aboriginal Australians (*22*), White Mountain Apaches (*23*) and Navajos in the southwestern United States (*24*), Maoris of New Zealand (*25*), and the bedouins of Israel (*26*).

Our data demonstrate that use of PCV7 in Alaska and northern Canada led to marked decreases in the incidence of IPD and PCV7 serotype–specific disease overall (all age groups combined) among indigenous persons (all age groups combined) and among indigenous children <2 years of age. Use of this vaccine has resulted in a near equalization of rates of PCV7 serotype–specific disease among indigenous children <2 years of age in Alaska and northern Canada. Our data also show decreases in disease among persons >2 years of age who were not targeted to receive vaccine. Although some children >2 years of age may have received vaccine as part of vaccine catch-up programs, older children and adults did not receive the vaccine. Our data show decreases in PCV7 serotype–specific disease among persons 20–64 years of age in northern Canada and among persons >65 years of age in Alaska. These data are consistent with decreasing rates of vaccine-type IPD among adults in the general US population (*27*).

The increased rate of non-PCV7–serotype disease in Alaska after introduction of PCV7, primarily among indigenous persons, is concerning. Increases of such magnitude have not been observed among the general US child population or elsewhere. However, continued vigilance is critical to monitor trends in pneumococcal disease and serotype distribution.

Incidence rates increased among children <2 years of age in 2 of 4 ICS members countries not using PCV7 over the study period (Norway and Finland). Norway began routine use of the vaccine among children in 2006. Continued collection of surveillance data will be critical in the coming years to assess the effect of pneumococcal vaccine, serotype shifts, and changes in antimicrobial drug susceptibility patterns.

Our data show that several serotypes (4, 6, 7, and 14) are common in northern circumpolar countries. Although serotype 1 was the most prevalent serotype in Greenland and northern Canada, it was not common in other circumpolar countries. Among countries that were not using PCV7 during the study period, ≈50% of all IPD cases in children <2 years of age were vaccine preventable (caused by serotypes present in PCV7). Use of 13-valent conjugate vaccine (which includes all PCV7 serotypes plus serotypes 1, 3, 5, 6A, 7F, and 19A) currently being evaluated for use in the United States could theoretically have prevented ≈70% of IPD cases among children <2 years of age.

In the 2 countries currently using PCV7 (Alaska and northern Canada), the proportion of penicillin-nonsusceptible isolates decreased. Published data on antimicrobial drug resistance in Alaska, the United States, and Canada showed an increasing proportion of *S*. *pneumoniae* drug-resistant isolates before vaccine implementation (*15*,*28*–*30*; www.cdc.gov/ncidod/aip/research/ar.html). Our data show a rapid decrease in the proportion of isolates resistant to penicillin and other antimicrobial drugs after PCV7 implementation. In the only 2 countries for which data on antimicrobial drug susceptibility were available and which were not using the vaccine (Iceland and Finland), the proportion of isolates resistant to penicillin increased over the study period.

To our knowledge, the ICS collaboration and data presented in this report are the first population-based assessment of IPD in the Arctic using similar case definitions and comparable laboratory methods. However, this study has several limitations. We did not collect detailed clinical and demographic information beyond what was available from medical record review and thus could not evaluate an extensive range of risk factors. Data on clinical findings, antimicrobial drug susceptibility, and risk factors are not collected consistently across the entire ICS network, and data on race/ethnicity are collected from only 2 ICS member countries. Because not all ICS member countries joined the network in the same year, the number of years of data available during the study period varied by country. In addition, limited information exists on diagnostic culturing practices of ICS member countries, which may lead to detection bias (milder cases more likely to be detected in some regions). This limitation may contribute to the wide variation in case-fatality rates across the ICS network. Finally, these data do not represent a complete picture of pneumococcal invasive disease in the far north because the Russian Federation does not participate in ICS, and data from only 1 region in northern Sweden are included.

The ICS project provides a broad view of IPD and the utility and status of prevention efforts in the Arctic. Demonstration of the effectiveness of PCV7 in Alaska and Canada and identification of issues relevant for future vaccine development are critical for decision making. Surveillance data on serotype and antimicrobial drug susceptibility distribution in Arctic countries provide necessary information for assessing the potential effect of current and future pneumococcal vaccines. Continuing evaluation of IPD in the ICS network will provide data necessary to maximize IPD prevention efforts throughout the region.
